# A modified arginine-depleting enzyme NEI-01 inhibits growth of pancreatic cancer cells

**DOI:** 10.1371/journal.pone.0231633

**Published:** 2020-04-30

**Authors:** Jeremy P. H. Chow, Yijun Cai, Daniel T. L. Chow, Steven H. K. Chung, Ka-Chun Chau, Ka-Ying Ng, Oscar M. Leung, Raymond M. H. Wong, Alan W. L. Law, Yu-On Leung, Sui-Yi Kwok, Yun-chung Leung

**Affiliations:** 1 New Epsilon Innovation Limited, Hong Kong, China; 2 State Key Laboratory of Chemical Biology and Drug Discovery, Department of Applied Biology and Chemical Technology and Lo Ka Chung Research Centre for Natural Anti-Cancer Drug Development, The Hong Kong Polytechnic University, Hong Kong, China; Columbia University, UNITED STATES

## Abstract

Arginine deprivation cancer therapy targets certain types of malignancies with positive result in many studies and clinical trials. NEI-01 was designed as a novel arginine-depleting enzyme comprising an albumin binding domain capable of binding to human serum albumin to lengthen its half-life. In the present work, NEI-01 is shown to bind to serum albumin from various species, including mice, rat and human. Single intraperitoneal administration of NEI-01 to mice reduced plasma arginine to undetectable level for at least 9 days. Treatment of NEI-01 specifically inhibited cell viability of MIA PaCa-2 and PANC-1 cancer cell lines, which were ASS1 negative. Using a human pancreatic mouse xenograft model, NEI-01 treatment significantly reduced tumor volume and weight. Our data provides proof of principle for a cancer treatment strategy using NEI-01.

## Introduction

Arginine is a nonessential amino acid for human cells as it can be generated through the urea cycle by argininosuccinate sythethase (ASS1) and argininosuccinate lyase (ASL) in normal cells. However, some tumor cells are deficient in ASS1, which can result in arginine auxotrophy.

Arginine deiminase (ADI) is a bacterial enzyme that catalyzes the hydrolysis of arginine to citrulline and ammonia. It has been reported that ADI inhibits the growth of several ASS1-negative tumors, such as melanoma and hepatocellular carcinoma (HCC) [1, 2], suggesting that it has potential as an anti-cancer agent. In fact, several chemically modified arginine-depleting enzymes are in different phases of clinical trials [3–6].

The *in vivo* application of arginine-depleting enzymes face two major problems: short circulating half-life in plasma and high immunogenicity [7]. One strategy for overcoming these problems is the covalent linkage of a well-known chemical modifier, polyethelene glycol (PEG), to the amino group of the proteins, providing a number of advantages such as low antigenicity, low toxicity and extended circulating half-life [8]. The modification has been used on at least three FDA approved proteins: PEG-asparaginase, PEG-adenosine deaminase and PEG-interferon-α-2b, which are used in various treatments. Recently, PEGylated ADI has been reported to enhance its potency as an anti-tumor drug as well as significantly lengthen its serum half-life [9, 10]. However, it has also been reported that long exposure to PEGylated proteins can still induce antibodies against PEG [11], which in turn decreases the circulation time of the proteins. Another disadvantage is that PEGylation may affect the accessibility of the substrates to the enzymes and thus reducing their specific activities [10].

Another strategy to extend the circulation time of therapeutic proteins is to genetically fuse them with an antibody Fc domain or serum albumin. Proteins containing such domains taken up by the cells will bind to Fc receptors (FcRn), which prevents lysosomal degradation and recycles back to the blood plasma [12]. By such modification, the circulating half-life can be extended in the range of weeks in humans.

NEI-01 is an enzyme that catalyzes the conversion of arginine to citrulline and ammonia. It was designed as an anti-cancer agent, which functions by reducing arginine in the blood and binding to albumin to extend circulating half-life.

With no symptoms at the early stages, pancreatic cancer is extremely deadly. According to the American Cancer Society, for all stages of pancreatic cancer combined, the 1-year relative survival rate is 20%, and the 5-year rate is 7%. Pancreatic cancer is a relatively chemoresistant malignancy. The tumor microenvironment is made of various stromal cell types, forming stromal barriers that prevent penetration of therapeutic agents into the tumor [13]. On the other hand, the metabolic reprogramming in most pancreatic cancers provides a rationale for amino acid deprivation as a therapeutic strategy [14]. It has been reported that pancreatic tumors and pancreatic cancer cell lines are frequently deficient in ASS1 [15].

In the present study, we described the production and purification of NEI-01. Purified NEI-01 exists as a dimer (~105 kDa) and is capable of binding to human serum albumin. NEI-01 was fully active and lowered arginine levels in mouse plasma to an undetectable level for at least 9 days, indicating an extended circulating half-life. Cancer cell lines with low expression levels of ASS1 were found to be sensitive to NEI-01 treatment. Using a human pancreatic mouse xenograft model, we showed that NEI-01 inhibited the tumor growth in a dose-dependent manner.

## Materials and methods

### Ethics statement

The mice with free access to food and water were maintained under pathogen-free conditions in Centralized Animal Facilities, The Hong Kong Polytechnic University according to the institutional guidelines. The Hong Kong Polytechnic University ethics board reviewed the study protocol, and they approved this research.

### Materials

All reagents were obtained from Sigma-Aldrich (St. Louis, MO) unless stated otherwise.

### DNA constructs

NEI-01-encoding plasmid regulated under IPTG induction was obtained by using GenScript's Gene synthesis service.

### Expression and purification of NEI-01

*E*. *coli* BL21 derivative (C3013I) were transformed with NEI-01-encoding plasmid regulated under IPTG induction. Four colonies were inoculated individually. Expression of NEI-01 was induced and confirmed with SDS-PAGE electrophoresis and activity assay. One of the colonies with highest expression and activity was selected for making bacterial stock and stored in small aliquots. For the seed culture, one aliquot was inoculated into 50 ml of seeding medium (containing 1.50 g of yeast extract and 0.25 g of NaCl) with kanamycin (50 μg/ml) and grown at 30°C for 16 h with continuous shaking at 250 rpm. The seed culture was then added to 1.25 L of medium (pH 7.4, containing 10 g of yeast extract, 15 g of tryptone, 8.38 g of Na_2_HPO_4_, 4.20 g of KH_2_PO_4_, 3.01 g of (NH_4_)_2_SO_4_, 12.50 g of glycerol, 1.25 g of glucose, 0.63 g of MgSO_4_·7H_2_O, 5 mg of Thiamine-HCl and 1 mM CaCl_2_) supplement with trace element in the BIOSTAT fermentor system (Sartoris, Germany) and grown at 28°C. Until the OD_600_ of the culture reached ~20, IPTG was added to a final concentration of 0.4 mM. The culture was further incubated for 16 h. During incubation, 500 ml of feeding medium (pH 7.4, containing 11.5 g of yeast extract, 18.5 g of tryptone, 2.41 g of NH_4_Cl, 0.67 g of (NH_4_)_2_SO_4_, 20 g of glycerol and 2 g of MgSO_4_·7H_2_O) supplement with trace element were applied at 0.5 ml/min. Aeration was regulated by varying the speed of stirring from 500 rpm to 2000 rpm to maintain 20% of air saturation.

NEI-01 was purified as described in Furaya *et al* with modification [16]. The bacteria were harvested by centrifugation. The cell pellet was resuspended in 400 ml of 20 mM of phosphate buffer (pH 7.4, containing 0.5 M NaCl). The cells were disrupted with APV-2000 homogenizer (SPX FLOW Inc, CA) and centrifuged. The pellet was washed 2 times with 140 ml of deionized water. After centrifugation, the inclusion bodies were solubilized with 40 ml of unfolding buffer (50 mM Tris pH 8.5, 6 M Guanidine-HCl). For protein refolding, the solution was added dropwise into 1000 ml of refolding buffer (20 mM Tris pH 7.2, 350 mM Glucose, 1 mM EDTA, 1 mM DTT) and incubated for 48 h. To remove the precipitate after refolding (clarification), the solution was filtered by Millistak+ Pod COHC system (Merck Millipore, Burlington, MA). The permeate was subjected to ion exchange chromatography using Q Sepharose Fast Flow column. The column was washed with 6 CV of wash buffer (50 mM Tris pH 7.4) and eluted using a 3 CV linear gradient from 0 to 100% of elution buffer (50 mM Tris, pH 7.4, 0.5 M NaCl). Fractions with NEI-01 were pooled together and subjected to hydrophobic interaction chromatography using a Phenyl FF column. Briefly, the column was conditioned with conditioning buffer (50 mM sodium phosphate, pH 7.4, 2 M ammonium sulfate). The pooled fraction was diluted with 2 part of Phenyl FF binding buffer (175 mM sodium phosphate, pH 7.4, 75 mM ammonium sulfate). The diluted protein was eluted with ~6 CV of Phenyl FF elution buffer (10 mM sodium phosphate, pH 7.4). A Sartobind STIC PA filter was used for endotoxin removal. Buffer exchange and concentrating process were performed with diafiltration buffer (10 mM sodium phosphate, pH 7.4, 140 mM sodium choride) by a tangential flow filtration (TFF) system (Merck Millipore). Bioburden filtration was performed by passing through a Sartopore Capsule 0.2 μm filter (Sartorius) (Purified NEI-01).

### Cell culture

The human hepatocellular carcinoma cell line SMMC7721 was obtained from Thermo Fisher Scientific (Waltham, MA). The human pancreatic carcinoma cell lines MIA PaCa-2 and PANC-1 were obtained from ATCC (Manassas, VA). Cells were propagated in RPMI1640 (for SMMC7721) or Dulbecco's modified Eagle's medium (DMEM) (for MIA-Paca-2 and PANC-1) supplemented with 10% (v/v) fetal bovine serum (for SMMC7721 and PANC-1) or 10% (v/v) fetal bovine serum and 2.5% (v/v) horse serum (for MIA PaCa-2) and 50 U/ml penicillin-streptomycin in a humidified incubator at 37°C in 5% CO_2_. The culture medium and serum were obtained from Invitrogen (Carlsbad, CA).

### Cell viability assay

Cell viability was assessed using a MTT assay obtained from Promega (Fitchburg, WI) according to the manufacturer's instruction. Briefly, cells (2000 cells / well) were seeded onto a 96-welll culture plate 16 h prior to NEI-01 treatment at designated concentrations (range from 0.001 mg/ml to 10 mg/ml). After 72 h, the culture media were replaced with 100 μl of media containing MTT (0.5 mg/ml) and further incubated for 4 h. Colorimetric development was then performed by adding 50 μl of DMSO and incubated at 37°C for 10 min. Experiments were repeated three times, and data represented as the mean of triplicate wells ± SEM.

### Protein concentration determination

The protein concentration was determined using Pierce BCA Protein Assay Kit (Thermo Fisher Scientific) according to the manufacturer's instruction.

### Enzymatic activity assay

The enzymatic activity of NEI-01 was assayed by colorimetric determination of the reaction product, citrulline [17]. Briefly, the reaction mixture (100 mM potassium phosphate pH 7.4, 20 mM L-ariginine and 5 μl enzyme solution in a final volume of 100 μl) was incubated at 37°C for 5 min. The reaction was terminated by adding an equal volume of 50% (w/v) trichloroacetic acid solution (TCA). The amount of citrulline was determined with diacetyl-monoxime. The specific activity of NEI-01 (U/mg) defines the amount of NEI-01 required to converts 1 μmole of L-arginine to 1 μmole of L-citrulline and 1 μmole of ammonia per min at pH 7.4 at 37°C per mg of protein.

### Animal

BALB/c female athymic nude mice (4-6-week-old) were purchased from Laboratory Animal Services Centre, The Chinese University of Hong Kong. The mice had free access to food and water and were maintained under pathogen-free conditions in the Centralized Animal Facilities, at the Hong Kong Polytechnic University according to the institutional guidelines (15-16/19-ABCT-R-CRF).

### Tumor xenograft

MIA PaCa-2 cells (5 x 10^6^) suspended in 100 μl PBS containing 50% (v/v) Matrigel matrix (BD Biosciences, San Jose, CA) were subcutaneously implanted into the flank of the nude mice. When the average tumor volume reached ~100 mm^3^, the mice were randomly divided into three groups and intraperitoneally administrated (twice a week) with PBS, 2 U or 5 U of NEI-01 per mouse. Tumors were measured using a Vemier caliper and the volume was calculated according to the formula: π/6 x length x width^2^. Mouse body weight was measured every week. At the end of the experiment, the tumors were excised from the sacrificed mice and weighted.

### Arginine and citrulline levels in plasma

For blood collection, a conscious mouse was restrained in a restraint tube. Its hind leg was immobilized. Petroleum jelly was applied to the caudal surface of the thight. The saphenous vein was punctured with a 25G needle. Drops of blood were collected using a lithium-heparin blood-gas capillary and transferred to a centrifugation tube. About 100 μl of blood was collected and centrifuged at 6000 rpm at 4°C for 10 min. The supernatant (plasma) was aspirated. Plasma (30 μl) was mixed with 20 μl of PBS with 50 μl of 10% sulfosalicylic acid (SSA) (w/v). The mixture was incubated at 4°C overnight. To remove the protein precipitate, the mixture was centrifuged at 15000 rpm at 4°C for 10 min. The sample was mixed with lithium loading buffer, filtered with a 0.45 μm filter and analyzed using 30+ Amino Acid Analyzer according to the manufacturer's instruction (Biochrom, Cambridge, UK).

### Antibodies and immunoblotting

Antibodies against ASS1 (ab170952), ASL (ab201026), Beclin 1 (ab210498), LC3B (ab51520), OTC (ab203859) and PARP1 (ab194586) were obtained from abcam (Cambridge, UK). Antibodies against β-ACTIN (MA515730) were obtained Thermo Fisher Scientific. Antibodies against AMPKα (2532), Phospho-AMPKα (Thr172) (2535S) and GAPDH (5174S) were obtained from Cell Signaling (Danvers, MA). Immunoblotting was performed as previously described [18].

### Statistical analysis

Data is presented as the mean ± SD of at least three independent experiments performed in triplicate. The statistical significance of differences was evaluated by the Student's t-test using GraphPad Prism 8 software. The *p* value less than 0.01 (*) was considered to be significant.

## Results

### Expression and purification of NEI-01

**Fig 1:**
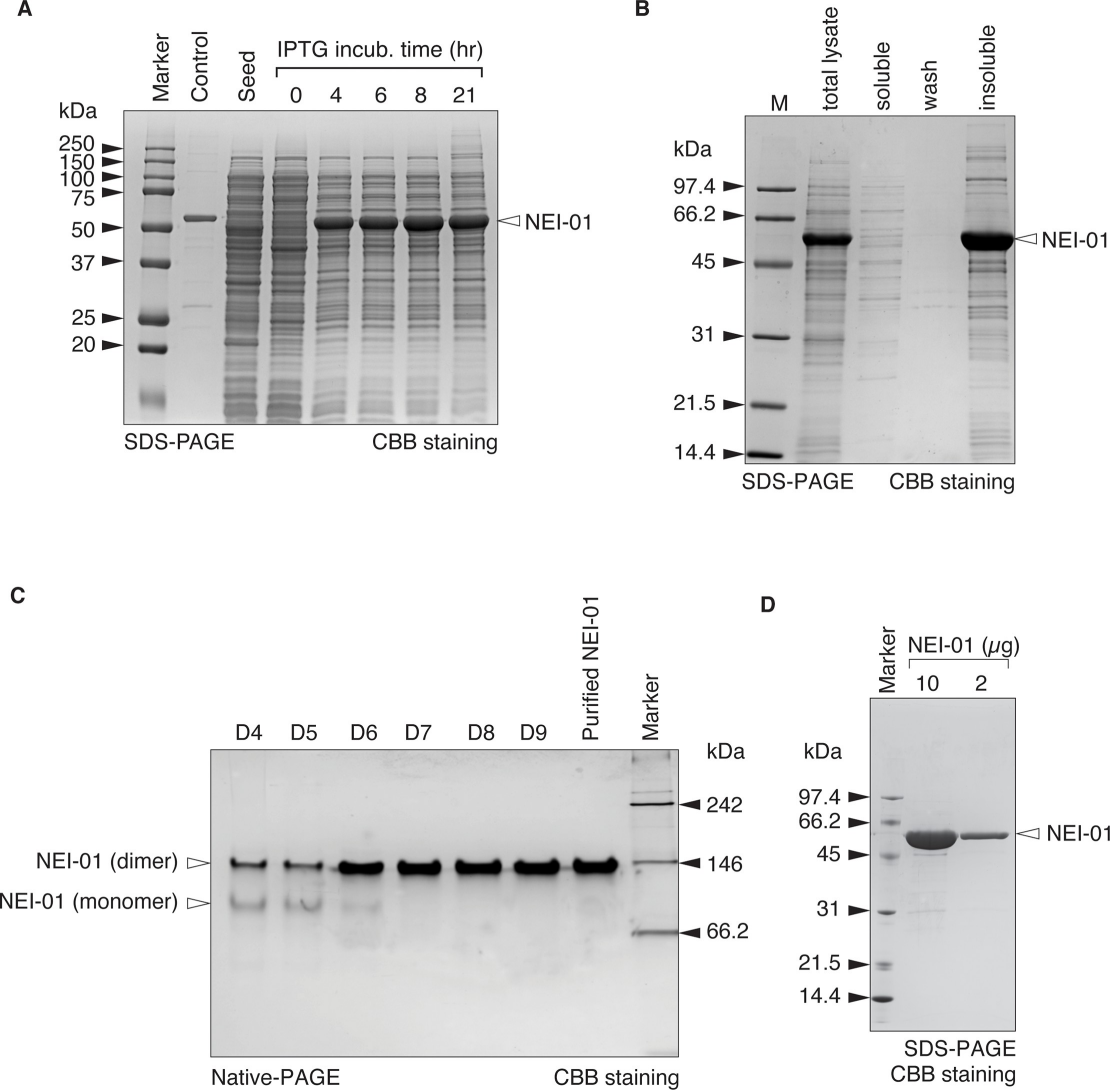
Expression and purification of NEI-01. (A) Expression of NEI-01 by IPTG induction. C3013I cells inducibly expressing NEI-01 were incubated with IPTG. Cells were harvested at indicated time points after induction and subject to SDS-PAGE followed by CBB staining. Each lane was normalized by cell density. The band representing NEI-01 was indicated with closed arrow head. (B) Expression of insoluble NEI-01. Harvested cell pellets were resuspended by phosphate buffer and disrupted by sonication on ice. The total cell lysate was fractionated into soluble, wash and insoluble fractions. The fractions were then subject to SDS-PAGE followed by CBB staining. The band representing NEI-01 was indicated with closed arrow head. (C) Purification of NEI-01. Purity of NEI-01 in different purification steps (D4- purified NEI-01) were analyzed by Native-PAGE. D4, Refolding; D5, Depth Filter; D6, QFF; D7, Phenyl FF; D8 STIC; D9, TFF. Bands representing NEI-01 in dimer and monomer forms were indicated with closed arrow head. (D) Purified NEI-01. Indicated amounts of purified NEI-01 were subject to SDS-PAGE followed by CBB staining. The gel was scanned by ChemiDoc imaging system and the lane percentage was analyzed by image lab software.

Plasmids with the inducible gene expressing NEI-01 were introduced into *E*. *coli* BL21 derivative (C3013I) for protein expression. The recombinant protein was ~53 kDa (monomer) in size. To optimize the protein expression, we first determined the concentration of IPTG in a mini-induction. The cells were grown in 2 ml of 2XYT medium at 37°C until the absorbance of OD_600_ reached ~0.8. Expression of NEI-01 was initialized by addition of IPTG to a final concentration of 0.1, 0.2, 0.4 or 0.8 mM of IPTG at 37°C. Samples were taken at indicated time points and subject to SDS-PAGE analysis (S1 Fig). The highest expression of NEI-01 was observed when incubated with 0.4 mM IPTG. Increasing the IPTG concentration to 0.8 mM did not result in a higher expression of NEI-01. A time course study was further performed with 0.4 mM IPTG in a 200 mL-scale. As shown in Fig 1A, the expression of NEI-01 was detected as early as 4-hours post-induction and peaked at 8 h, contributing more than 30% of the total protein. In these optimized conditions, NEI-01 was expressed in insoluble form (Fig 1B).

According to Furaya *et al*, insoluble ADI was dissolved in phosphate buffer supplemented with 5 M Guanidine-HCl, 1 mM EDTA and 5 mM DTT [16]. However, we found that phosphate buffer supplemented with 6 M guanidine-HCl only worked in our case. We next tried to optimize the refolding buffer mentioned in Furaya *et al* by supplementation of several additives, including arginine, glucose, KCl and PEG 200. The enzymatic activity of NEI-01 refolded with the buffer supplemented with different additives was measured by activity assay in term of U/ml. The result showed that increasing the concentration of glucose in the refolding buffer improved the activity of NEI-01 (S2 Fig). On the other hand, other additives did not improve the NEI-01 yield as mentioned. The difference may be because of the origin of ADI mentioned in Furaya *et al* is from *Mycoplasma hominis* while our modified ADI is from *Mycoplasma arginini* [16].

To achieve a higher purity, multiple purification steps were performed (see Materials and Methods). Briefly, the bacterial cell paste was resuspended in phosphate buffer for homogenization. The soluble proteins in the bacterial cell lysate were removed by centrifugation. The insoluble fraction (inclusion bodies) was solubilized and unfolded with unfolding buffer. The protein was then refolded by dilution with and incubated for 72 h (protein refolding, D4 step). The precipitates formed in the process were removed by filtration (clarification, D5 step). The permeate was subjected to ion exchange chromatography using a Q Sepharose Fast Flow column (D6 step) and then further purified using hydrophobic interaction chromatography (HIC) on a Phenyl FF column (D7 step). Removal of endotoxin was accomplished by Sartobind STIC PA filtration (D8 step). Buffer exchange and concentration were performed using a TFF system (D9 step). Finally, bioburden filtration was performed using Sartopore Capsule 0.2 μm (Purified NEI-01). The purity of samples from the purification steps (D4-D9 steps) and purified NEI-01 were compared using native-PAGE analysis. Despite the major band in steps D4 and D5 representing the active dimeric form of NEI-01 with expected molecular weight, monomer was also detected (Fig 1C), suggesting the presence of misfolded NEI-01 during the protein refolding process. The monomeric form was almost completely removed after HIC (D7 step). As shown in Fig 1D, 10 μg and 2 μg of NEI-01 were subjected to SDS-PAGE followed by densitometric analysis. Using a ChemiDoc MP system with Image Lab software, the purity of NEI-01 was determined to be >95%. The specific activity of the purified NEI-01 was about 38–60 U/mg.

#### Binding of NEI-01 with human serum albumin

**Fig 2:**
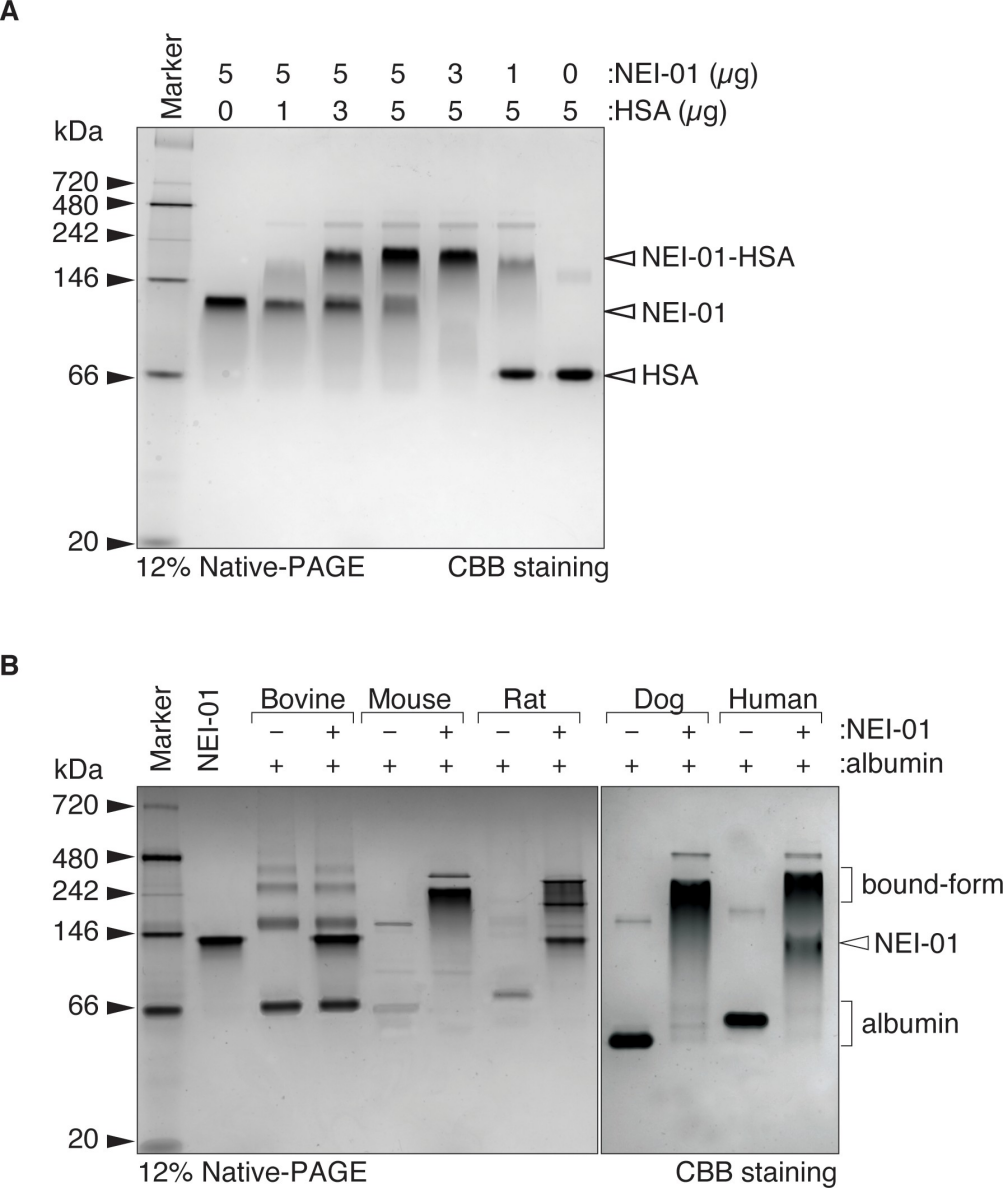
Binding of NEI-01with human serum albumin. (A) Gel shift assay for NEI-01 binding to human serum albumin (HSA). Indicated amounts of NEI-01 were incubated with different amounts of HSA and subject to Native-PAGE followed by CBB staining. The bands representing native states of NEI-01 (~104 kDa, dimer), HSA (~66 kDa) and NEI-01-HSA (~236 kDa bound-form) were indicated with closed arrow heads. (B) Binding of NEI-01 with serum albumins of different species. As a control, NEI-01 was incubated without albumin. Albumins from bovine, mouse, rat, dog or human were incubated with or without NEI-01 in 1:1 ratio. The samples were then subject to Native-PAGE followed by CBB staining. Bands representing albumin, NEI-01 and NEI-01-albumin bound-form were indicated with closed arrow head and closing parentheses.

We hypothesized that NEI-01 is capable of binding to human serum albumin (HSA), which prolongs its circulating half-life. To confirm the HSA binding, different ratios of NEI-01 and HSA were incubated at room temperature for 20 min and analyzed using gel shift assay [19]. As expected, NEI-01 exists in a dimeric form migrated with ~105 kDa in molecular weight (Fig 2A). After incubating with HSA, the migration of the NEI-01 was slowed down. The more HSA incubated with NEI-01, the more NEI-01 migrated with a larger molecular weight until HSA was in excess in a 5 to 1 ratio to NEI-01 (Fig 2A). The specificity of NEI-01 binding to serum albumin from different species was also tested. As shown in Fig 2B, NEI-01 bound to serum albumin from mouse, rat, dog and human but not from bovine. The specific activity of NEI-01 showed no difference with or without binding to HSA (Table 1).

**Table 1:** No change in NEI-01 enzymatic activity (U/mg) after binding to albumin.

	without HSA	with HSA	% change of activity
*NEI-01*	38.4	38.3	-0.4
*SD*	±0.41	±1.43	0.36

NEI-01 were incubated with or without HSA. After incubation, the samples were subjected to activity assays. Results from three independent experiments, their mean value and the percentage change of enzymatic activity were shown.

#### Pharmacodynamics and pharmacokinetics of NEI-01 in mice

**Fig 3:**
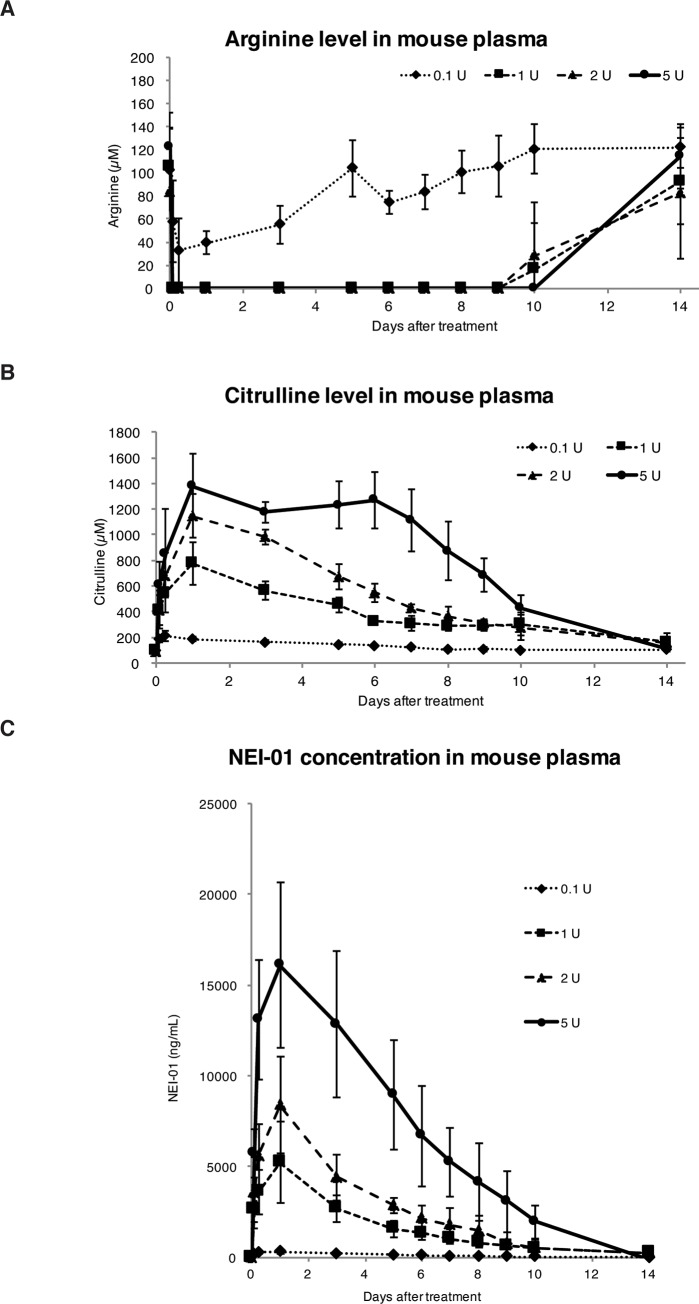
Pharmacokinetics and pharmacodynamics of NEI-01 in mice. Deprivation of arginine and increase in citrulline in mouse plasma after NEI-01 administration. Mice were intraperitoneal administrated (i.p.) with 0.1 U, 1 U, 2 U or 5U of NEI-01 at day 0. At day 0, bloods were collected at 1 hr prior to the administration as a control. Bloods were also collected at day 1, 2, 4, 5, 7 and 8. Plasma were prepared by centrifugation. After removal of protein by precipitation, the plasmas were subject to the amino acid analyzer for the measurement of arginine (A) and citrulline (B) levels. Data shown was the mean ± SD of 5 mice. (C) Time course of NEI-01 concentration in mouse plasma after NEI-01 administration. Mice (*n* = 6 for each group) were treated exactly as panel A except that bloods were collected at day 0.08 (~ 2 hr), 0.25 (~6 hr), 1, 3, 5, 6, 7, 8, 9, 10, 14. After plasma preparation, the samples were subject to ELISA using antibodies against NEI-01. The NEI-01 concentration of NEI01 in mouse plasma was shown as mean ± SD of triplicate wells.

NEI-01 is novel arginine-depleting enzyme that catalyzes the conversion of arginine to citrulline and ammonia. Administration of arginine deiminase to mice has been reported to reduce the level of arginine in the mouse blood stream. However, this reduction persisted for only 12–24 h after injection and the circulating half-life was about 4 h [20, 21]. NEI-01 was confirmed to bind to mouse serum albumin (Fig 2B). We then analyzed the pharmacodynamics and pharmacokinetics of NEI-01 in mouse. To evaluate the pharmacodynamics of NEI-01, mice were administrated a single dose of 0.1 U, 1 U, 2 U or 5 U intraperitoneally and their levels of plasma arginine and citrulline were determined. As shown in Fig 3A, concentrations of plasma arginine dropped from ~100 μM to undetectable levels after 2 h in single dose administrations of 1 U, 2 U or 5 U of NEI-01. The reduction persisted for at least 9 days and returned to normal levels within 14 days. Reciprocally, plasma citrulline levels in these mice was dramatically increased in a dose dependent manner and dropped back to normal level within 14 days (Fig 3B). For the pharmacokinetics, the concentrations of NEI-01 in mouse plasma were quantified by ELISA (Fig 3C). NEI-01 was detectable at 2 h after administration and peaked at 1 day. Correlatively, the concentration of NEI-01 in the plasma showed similar patterns to the results from pharmacodynamics study.

#### Growth inhibition of ASS1-negative cancer cells by NEI-01 treatment

**Fig 4:**
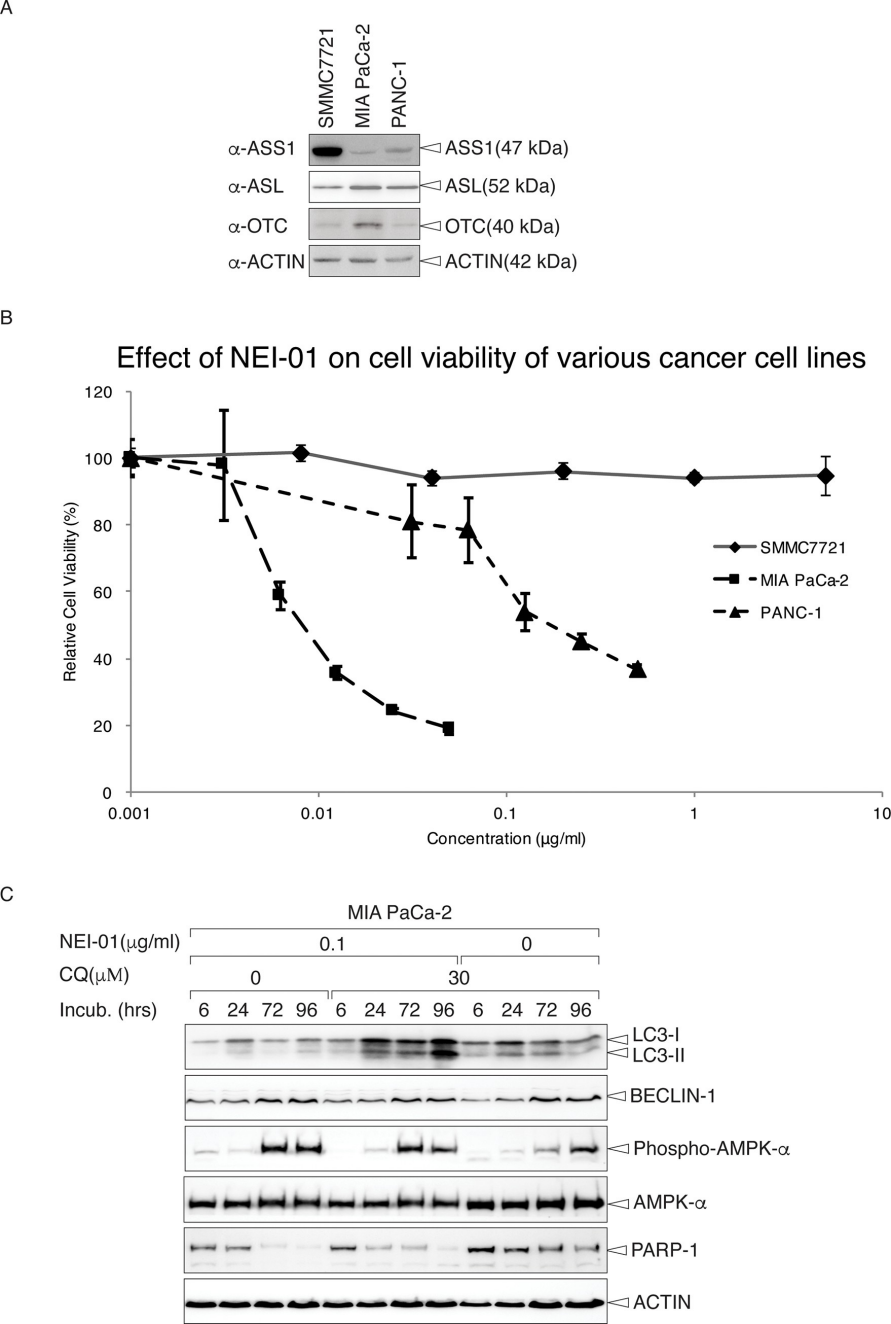
Expression of ASS1 in various cell lines and the effect of NEI-01 on their cell viability. (A) Expression of ASS1, ASL and OTC in SMMC7721, MIA PaCa-2 and PANC-1. Unperturbed asynchronously growing cells were harvested by trypsinization. After cell lysis, total cell lysates were subject to immunoblotting with anti-ASS1, anti-ASL, anti-OTC or anti-ACTIN antibodies. (B) Inhibition of cell growth of MIA PaCa-2 and PANC-1 cells but not SMMC7721 cells by NEI-01 treatment. SMMC7721 cells, MIA PaCa-2 cells or PANC-1 were seeded in 96 well-plate with ~3000 cells/well density 24 hr prior to NEI-01 treatments. After 72 hr, cells were subject to MTT assay. The relative cell viability in % vs the NEI-01 concentration was shown as mean ± SD of triplicate wells. (C) Activation of autophagic pathway upon NEI-01 treatment. MIA PaCa-2 cells were treated with indicated amount of NEI-01 for 3 or 7 days. The cells were then harvested and subject to immunoblotting using designated antibodies.

It has been previously reported that about 50% of cancer cells from patients lack ASS1, which are auxotrophic for arginine [22]. We first tested the expression of ASS1, ASL and OTC in SMMC7721, MIA PaCa-2 and PANC-1 using immunoblotting. In agreement with reported results [15, 23], SMMC7721 expressed high level of ASS1 while only weak bands (signals) were detected in MIA PaCa-2 and PANC-1 (Fig 4A). To confirm the effect of NEI-01 on their cell viability, cells were treated with different doses of NEI-01 for 72 h prior to MTT assay. As shown in Fig 4B, MIA PaCa-2 and PANC-1 cells were more sensitive to NEI-01. On the other hand, there was almost no effect of NEI-01 on SMMC7721 viability, further suggesting the strong correlation between the expression of ASS1 and sensitivity to NEI-01. The IC50 of these cells were calculated, showing that the effective concentration of NEI-01 to the cell viability of MIA PaCa-2 and PANC-1 cells was in the range of ng/ml (Table 2), indicating that NEI-01 is highly potent. It has been reported that arginine deprivation triggers atypical autophagic cell death via mitochondrial damage [31]. To confirm the reduction of cell viability by autophagic cell death, Mia PaCa-2 cells were treated with designated concentrations of NEI-01 with or without choloquine (CQ). At indicated time-points, cells were harvested and subjected to immunoblotting using antibodies against several autophagic and apoptotic markers. As shown in Fig 4C, the expression levels of LC3-II, BECLIN-1 and phospho-AMPKα were increased upon NEI-01 treatment, suggesting autophagy plays the role in NEI-01-induced cell death. On the other hand, the expressions levels of PARP-1 were decreased upon the treatment, showing the activation of apoptotic pathways. These results show that apoptosis and autophagy also play a role in the cell death mechanism.

**Table 2:** IC50 values of NEI-01 in various cell lines.

Cancer cell line	IC50 (ΜG/ML)
**SMMC7721**	>10
**MIA PACA-2**	0.086
**PANC1**	0.190

The IC50 of SMMC7721 cells, MIA PaCa-2 and PANC-1 cells were calculated from the MTT assay in (Fig 4B).

#### Anticancer activity of NEI-01 in human pancreatic mouse xenograft model

**Fig 5:**
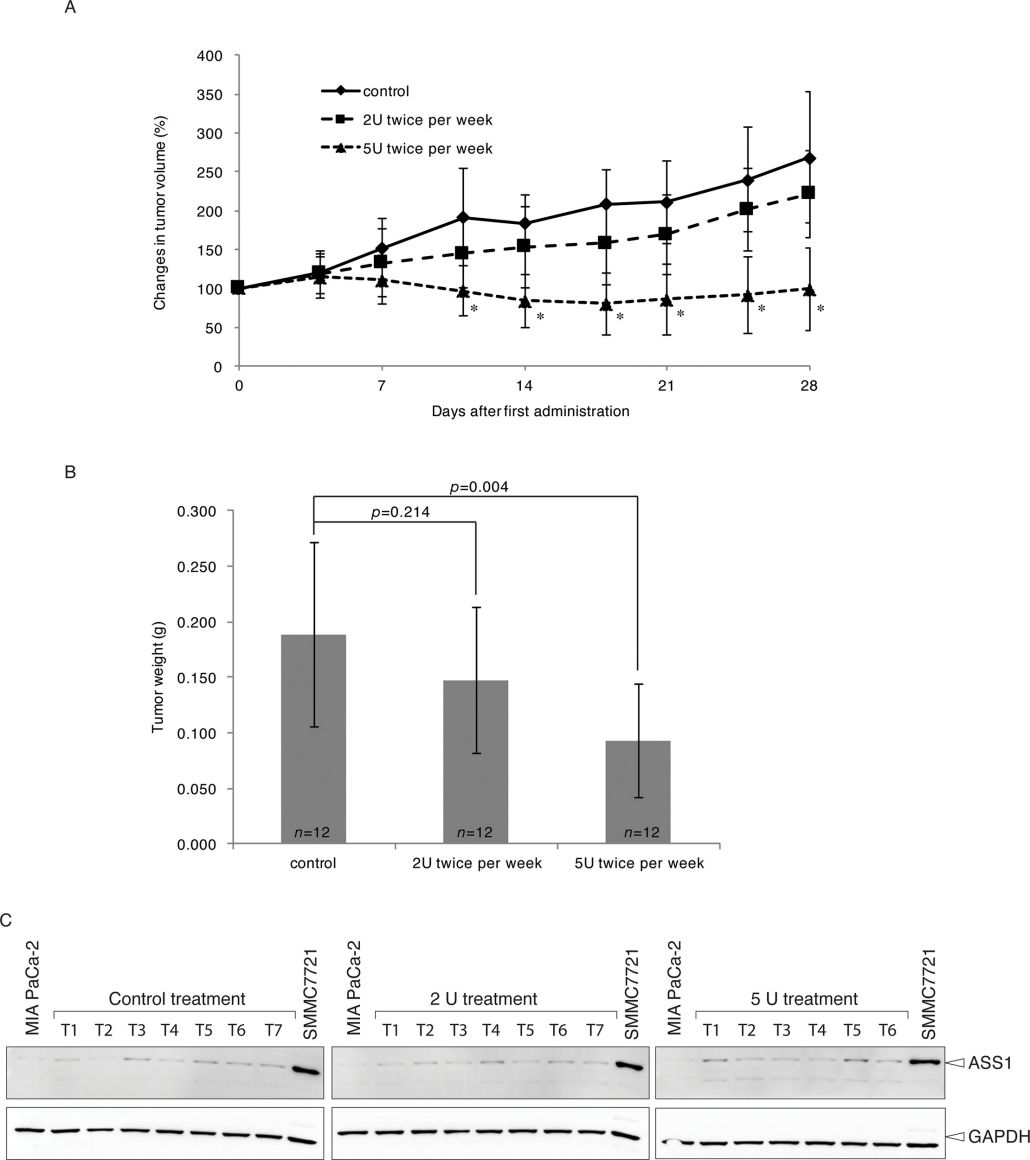
Anti-tumor efficacy of NEI-01 in human pancreatic mouse xenograft model. (A) Change in tumor volume of MIAPaCa-2 xenografts. Mice bearing subcutaneous MIA PaCa-2 xenograft were treated with buffer, 2U twice a week or 5U twice a week of NEI-01 as indicated. The volumes of the tumor were measured before each administration. Data shown as the mean ± SD of 12 mice and are representative of two independent experiments; *n* = 12, student's *t*-test,* *p*<0.01, Control vs 2U twice per week or 5 U twice per week. (B) Change in tumor weight of MIA PaCa-2 xenografts. Mice from (A) were sacrificed at day 28 after tumor volume measurement. The xenografts were isolated and weighted. Data shown as the mean ± SD of 12 mice and are representative of two independent experiments. The *p*-value of t-test were shown (*n* = 12, student's *t*-test, Control vs 2U twice per week or 5 U twice per week). (C) Expression of ASS1 in MIA PaCa-2 xenograft after NEI-01 treatment. The xenografts isolated from (B) were subject to immunoblotting using anti-ASS1 and anti-GAPDH antibodies.

The anticancer activity of NEI-01 was investigated *in vivo* using mouse xenograft model. MIA PaCa-2 cells were subcutaneously implanted into the flank of nude mice. When the average tumor volume reached ~100 mm^3^, the mice were randomized into three groups and intraperitoneally administrated (twice a week) PBS, 2 U of NEI-01 or 5 U of NEI-01. As shown in Fig 5A and 5B, tumor volume (*p* <0.01) and weight (*p* = 0.004) were significantly reduced by NEI-01 administration (5 U twice a week i.p.) compared to the control treatment. The tumors were randomly selected for detection of ASS1 expression using immunoblotting. Only a very slight increase in ASS1 expression was observed in all three groups (Fig 5C). The plasma arginine and citrulline levels before administration of NEI-01 and just before scarifying were also analyzed. The reduction of plasma arginine persisted over the course of the experiment (S3 Fig).

#### Re-expression of ASS1 in MIA PaCa-2 cells adapted with NEI-01

**Fig 6:**
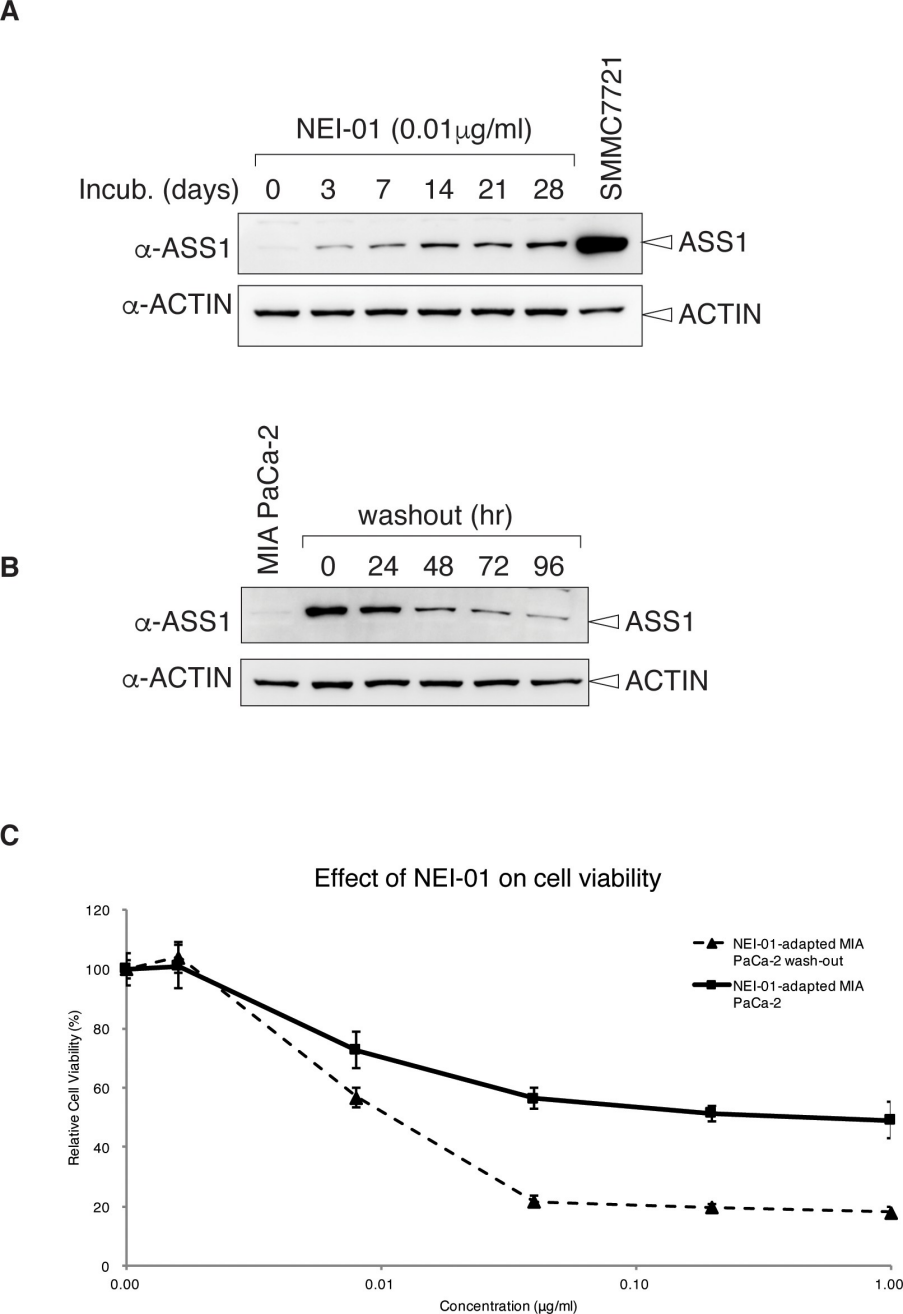
Expression of ASS1 upon long-term NEI-01 treatment. **(**A) Re-expression of ASS1 in NEI-01 treated MIA-PaCa-2 cells. MIA-PaCa-2 cells were propagated in DMEM supplemented with 0.01 μg/ml of NEI-01. Cells were harvested at indicated time points and subject to immunoblotting using anti-ASS1 and anti-ACTIN antibodies. Unperturbed asynchronously growing SMMC7721 cells were used as a control. (B) Reduction of ASS1 expression in NEI-01-adapted MIA PaCa-2 cells after removing of NEI-01 in the medium. MIA PaCa-2 cells adapted to NEI-01 from (A) were washed with PBS and grown in DMEM without NEI-01. The cells were harvested at indicated time points and subject to immunoblotting using anti-ASS1 and anti-ACTIN antibodies. (C) Resensitizing NEI-01-adapted cells to NEI-01. NEI-01-adapted MIA PaCa-2 cells from (A) and grown in medium without NEI-01 for 96 hr from (B) were seeded in 96 well-plate with ~3000 cells/well density 24 hr prior to NEI-01 treatments. After 72 hr, cells were subject to MTT assay. The relative cell viability in % vs the NEI-01 concentration was shown as mean ± SD of triplicate wells.

It has been reported that arginine-deprivation induces the re-expression of ASS1 and results in the resistance of its auxotrophic effect [24, 25]. To confirm this, MIA PaCa-2 cells were propagated in DMEM supplemented with 0.01 μg/ml of NEI-01. The expression of ASS1 was then analyzed at days 0, 3, 7, 14, 21 and 28 using immunoblotting method. As shown in Fig 6A, expression of ASS1 increased along with the incubation period and peaked at day 14. We finally established a NEI-01-adapted MIA PaCa-2 cell line with increased expression of ASS1 (Fig 6B). On the other hand, the expression of ASS1 in NEI-01-adapted MIA PaCa-2 cells reduced to a low level in 96 hr if NEI-01 was removed from the medium (Fig 6B). Not surprisingly, NEI-01-adapted cells resisted NEI-01 treatment in cell viability assays (Fig 6C). Interestingly, those cells released from NEI-01 treatment (NEI-01 washout) for 96 hr resensitized to NEI-01 treatment (Fig 6C) with a slightly increased IC50, 0.012 μg/ml.

## Discussion

In the present study, we found that single administration of NEI-01 depleted mouse plasma arginine to undetectable level for at least 9 days, showing an extended circulating half-life. The results demonstrated that NEI-01 inhibited cell viability of ASS1-negative cancer cell lines (e.g. MIA PaCa-2, PANC1). Using a human pancreatic mouse xenograft model, NEI-01 was shown to significantly reduce the tumor volume and weight.

Arginine deprivation cancer therapy targets a significant fraction of malignancies which are characterized by the loss of enzymes for urea cycle. ASS1, one of the key enzymes for urea cycle, catalyzes the condensation of aspartate and citrulline into argininosuccinate which is further processed to arginine. ASS1 deficiency has been identified in a broad spectrum of tumors. Even though its regulatory mechanism is not clearly understood, it is believed that expression of ASS1 was epigenetically regulated through aberrant methylation in the ASS1 promoter [26]. Studies have targeted certain types of sarcoma, melanoma, hepatocellular carcinoma, prostate cancer, leukemia, lymphoma, and pancreatic cancer with low expression of ASS1 and results showed that the treatment with pegylated form of arginine deiminase (ADI-PEG 20) leads to significant tumor growth inhibition [27–30].

To extend the circulating half-life of protein drugs, a chemical modifier polyethelene glycol (PEG) can be covalently linked to the amino group of the proteins. This solution provides advantages in several aspects but may affect the accessibility of the substrates to the enzymes and thus reduce their specific activities [10]. In a study, the catalytic turnover rate (Kcat) and substrate affinity (Km) of alpha-chymotrypsin was significantly altered after pegylation [31]. For NEI-01, binding to human serum albumin did not reduce its specific activity but still resulted in an extension of the circulating half-life of the protein and kept the plasma arginine to undetectable levels for at least 9 days. It has been reported that a pegylated form of ADI and arginase reduced plasma arginine to 8 days and 3 days, respectively in mice [10, 32]. In this regard, NEI-01 shows better performance.

Treatment of NEI-01 inhibited the growth of pancreatic cancer cells *in vitro* and *in vivo*. It has been shown that metabolic stress induced autophagy, however it is still controversial whether it promotes apoptotic cell death [33]. More specifically, arginine deprivation triggered atypical autophagic cell death via mitochondrial damage [34]. Although we observed LC3 phosphatidylethonalamine conjugation, accumulation of BECLIN-1 and phophorylation of AMPK-α upon NEI-01 treatment, PARP-1 was also degraded in a CQ-dependent manner (Fig 4C), suggesting the crosstalk of autophagy and apoptosis. Arginine deprivation has reportedly induced ER stress in solid cancer cells. Recently, the crosstalk of autophagy and apoptosis was shown to play the major role in this condition and may become a therapeutic strategy in cancer [35,36]. Further studies await the exploitation of combination therapy, such as with autophagy inhibitors.

Recently, several clinical studies have tested the effect of ADI-PEG 20 in difficult-to-treat cancers such as advanced pancreatic adenocarcinoma and advanced hepatocellular carcinoma (HCC). Lowery *et al*. have conducted a phase 1/1B trial of ADI-PEG-20 plus nab-paclitaxel and gemcitabine in patients with advanced pancreatic adenocarcinoma [37]. ADI-PEG 20 was well tolerated in combination with gemcitabine and nab-paclitaxel. Importantly, they found that the overall response rate among patients treated in the first-line setting was 45.5% (5 of 11). The median progression-free survival (PFS) time for these patients was 6.1 months and the median overall survival (OS) time was 11.3 months. More importantly, anti-cancer activity was observed in previously treated and untreated patients with advanced pancreatic cancer and in patients with ASS1-deficient and -proficient tumors. Interestingly, Singh *et al*. suggested that ADI-PEG 20 might be used to sensitize pancreatic cancer to radiotherapy via metabolic dysregulation [38]. They found that ADI-PEG 20 potently radiosensitized ASS1-deficient pancreatic cancer cells (MiaPaCa-2, Panc-1, AsPc-1, HPAC, and CaPan-1), but not ASS1-expressing cell lines (Bxpc3, L3.6pl, and SW1990). In their *in vivo* studies in two xenograft models, they observed significant tumor growth delays, which were associated with enhanced expression of ER stress proteins and apoptosis markers. It seems that ADI-PEG 20 can augment the effects of radiation by triggering the ER stress pathway. This leads to apoptosis in pancreatic tumor cells. On the other hand, Harding *et al*. reported the exciting results of a phase 1 study of ADI-PEG 20 and modified FOLFOX6 in patients with advanced HCC and other gastrointestinal malignancies [39]. They found that the median PFS and OS were 7.3 and 14.5 months, respectively. Moreover, arginine levels were depleted with therapy despite the emergence of low levels of anti-ADI-PEG 20 antibodies. Importantly, the data suggested that concurrent modified FOLFOX6 plus ADI-PEG 20 injected weekly shows an acceptable safety profile and favorable efficacy compared to historic controls. Furthermore, James *et al*. described the plan for the ongoing phase II study of ADI-PEG 20 and FOLFOX6 in patients with advanced HCC [40]. This is an international, multicenter, single-arm, open-label phase 2 trial of ADI-PEG 20 and FOLFOX6 for advanced HCC patients with Child-Pugh A liver function who progressed on ≥ 2 prior lines of prior systemic therapy.

In conclusion, NEI-01 depleted plasma arginine to undetectable levels and inhibited pancreatic cancer cell growth *in vitro* and *in vivo*. Therefore, NEI-01 may prove to be an effective treatment for pancreatic cancer.

## Supporting information

S1 Fig Optimization of the induction of NEI-01 expression by IPTG. C3013I cells inducibly expressing NEI-01 were incubated with 0.1 mM (upper left panel), 0.2 mM (upper right panel), 0.4 mM (lower left panel) or 0.8 mM (lower right panel) of IPTG. Cells were harvested at designated time points after induction and subjected to SDS-PAGE followed by CBB staining. Each lane was normalized by cell density. The band representing NEI-01 was indicated with closed arrow head.
(TIF)

S2 Fig Enhancement of refolding yield by adding glucose as addictive. Isolated inclusion bodies were solubilized with unfolding buffer as described in Materials and Methods. The unfolded protein was added dropwise into testing buffer (20 mM Tris pH 7.2, 1 mM EDTA, 1 mM DTT) with different concentrations of arginine (A), glucose (B), glycerol (C) or PEG 200 (D). After incubation for 48 hr, the samples were subjected to enzymatic activity assay. The relative activity in % vs the addictive concentration was shown.
(TIF)

S3 Fig Decrease in arginine and increase in citrulline after NEI-01 administration. The plasma from xenograft bearing mice from Fig 5 were subjected to the amino acid analyzer for the measurement of arginine (A) and citrulline (B) levels.
(TIF)

S1 Raw images (PDF)
