# Supplementary figures and images for: A modified arginine-depleting enzyme NEI-01 inhibits growth of pancreatic cancer cells
Source: PLoS One. 2020 Apr 30;15(4):e0231633. doi: 10.1371/journal.pone.0231633 (PMC7192632; doi:10.1371/journal.pone.0231633)

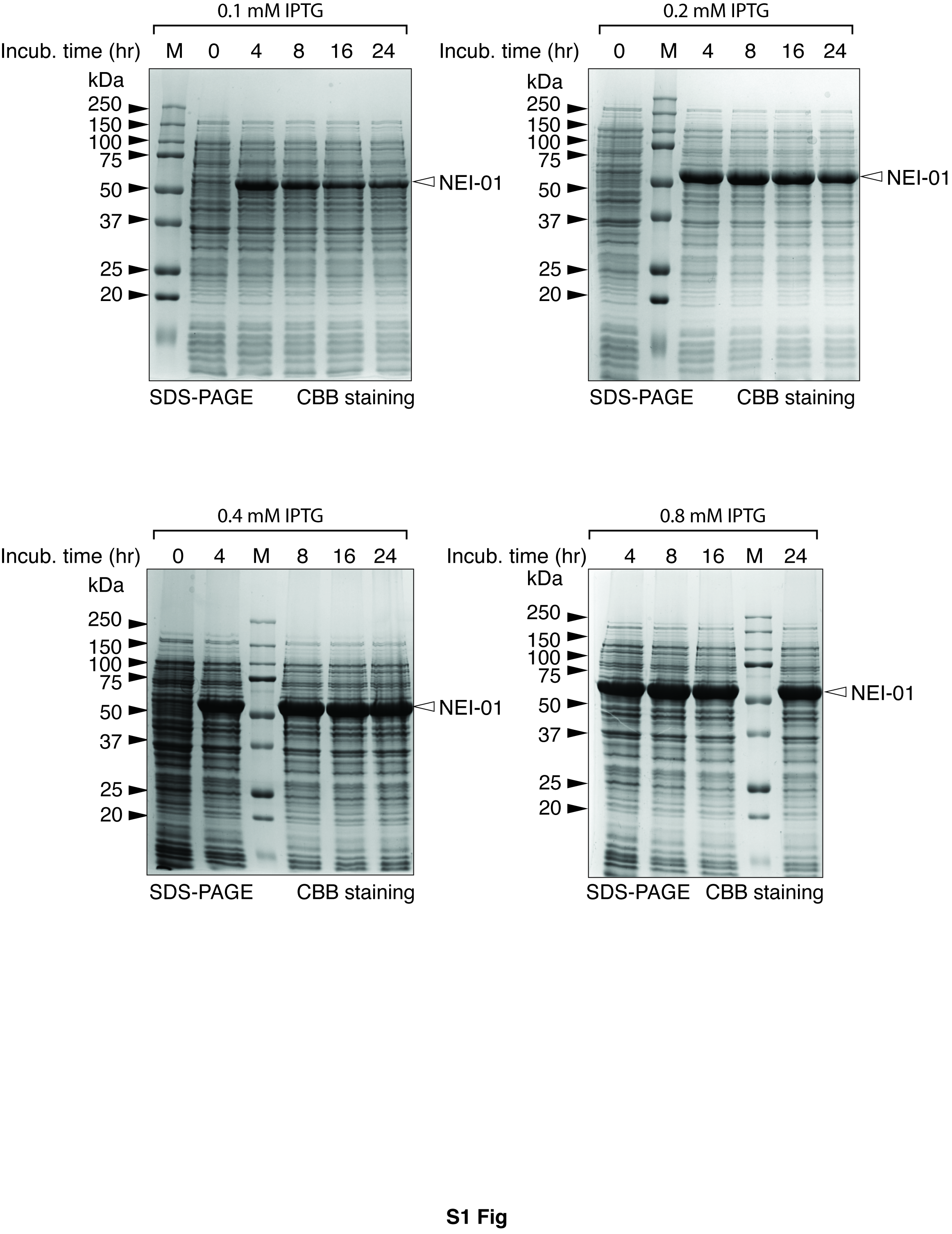

Supplement: S1 Fig — C3013I cells inducibly expressing NEI-01 were incubated with 0.1 mM (upper left panel), 0.2 mM (upper right panel), 0.4 mM (lower left panel) or 0.8 mM (lower right panel) of IPTG. Cells were harvested at designated time points after induction and subjected to SDS-PAGE followed by CBB staining. Each lane was normalized by cell density. The band representing NEI-01 was indicated with closed arrow head. (TIF) [file pone.0231633.s001.tif]

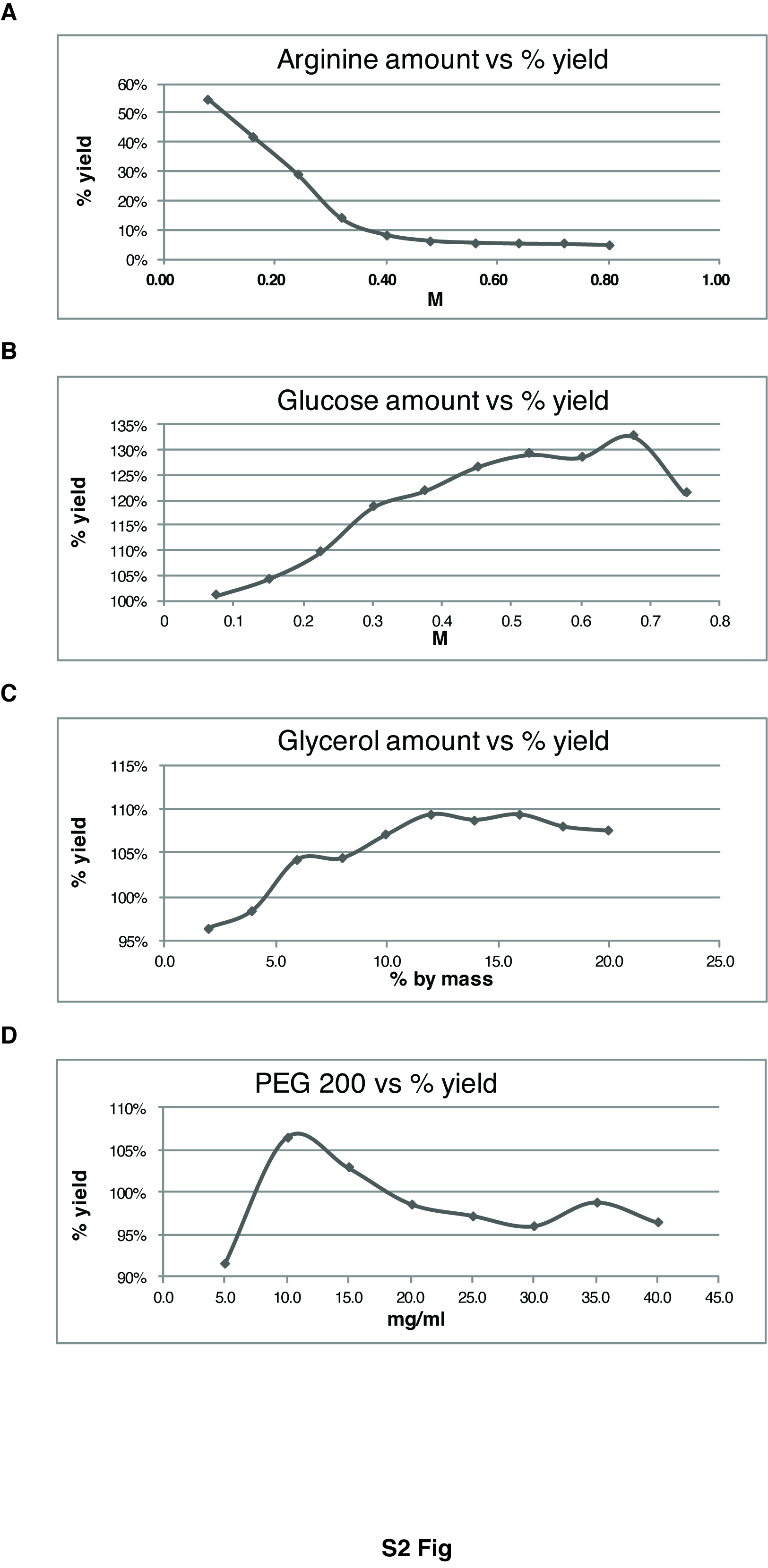

Supplement: S2 Fig — Isolated inclusion bodies were solubilized with unfolding buffer as described in Materials and Methods. The unfolded protein was added dropwise into testing buffer (20 mM Tris pH 7.2, 1 mM EDTA, 1 mM DTT) with different concentrations of arginine (A), glucose (B), glycerol (C) or PEG 200 (D). After incubation for 48 hr, the samples were subjected to enzymatic activity assay. The relative activity in % vs the addictive concentration was shown. (TIF) [file pone.0231633.s002.tif]

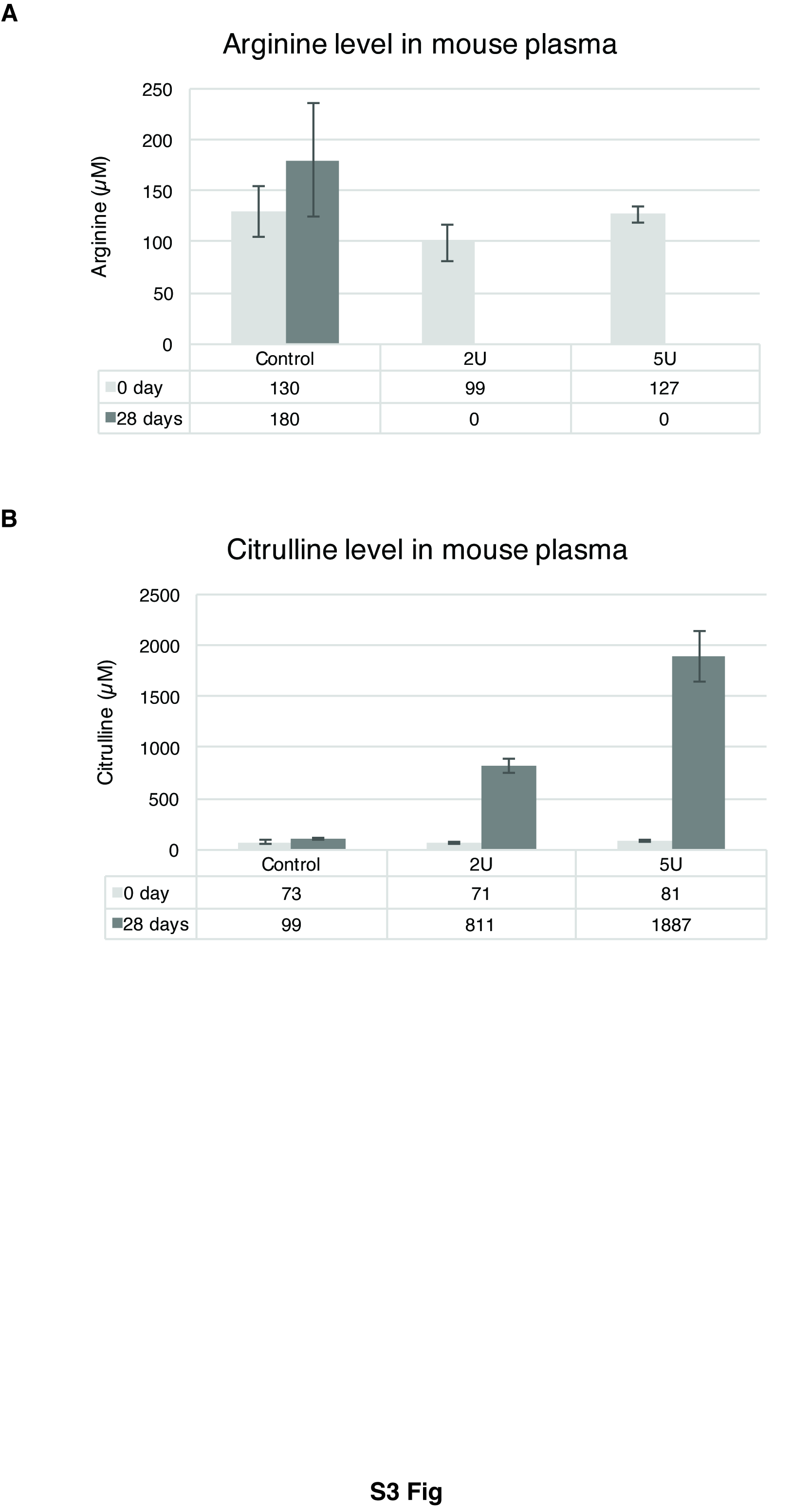

Supplement: S3 Fig — The plasma from xenograft bearing mice from Fig 5 were subjected to the amino acid analyzer for the measurement of arginine (A) and citrulline (B) levels. (TIF) [file pone.0231633.s003.tif]
