# Supplementary material for: A modified arginine-depleting enzyme NEI-01 inhibits growth of pancreatic cancer cells
Source: PLoS One. 2020 Apr 30;15(4):e0231633. doi: 10.1371/journal.pone.0231633 (PMC7192632; doi:10.1371/journal.pone.0231633)

Fig 4A

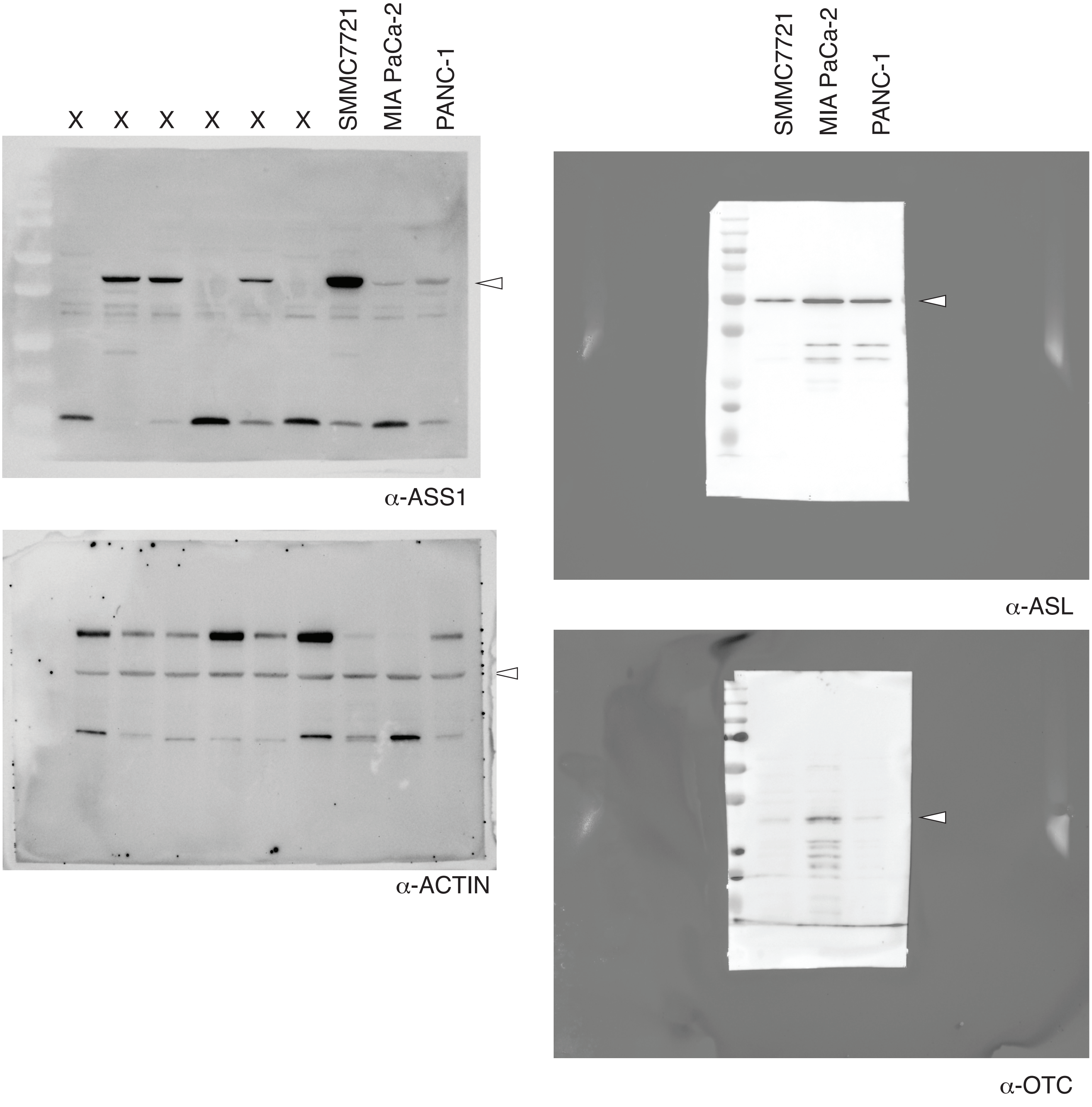
X
X
X
X
X
X
SMMC7721
MIA PaCa-2
PANC-1
α-ASS1
α-ACTIN
SMMC7721
MIA PaCa-2
PANC-1
α-ASL
α-OTC

Fig 4C

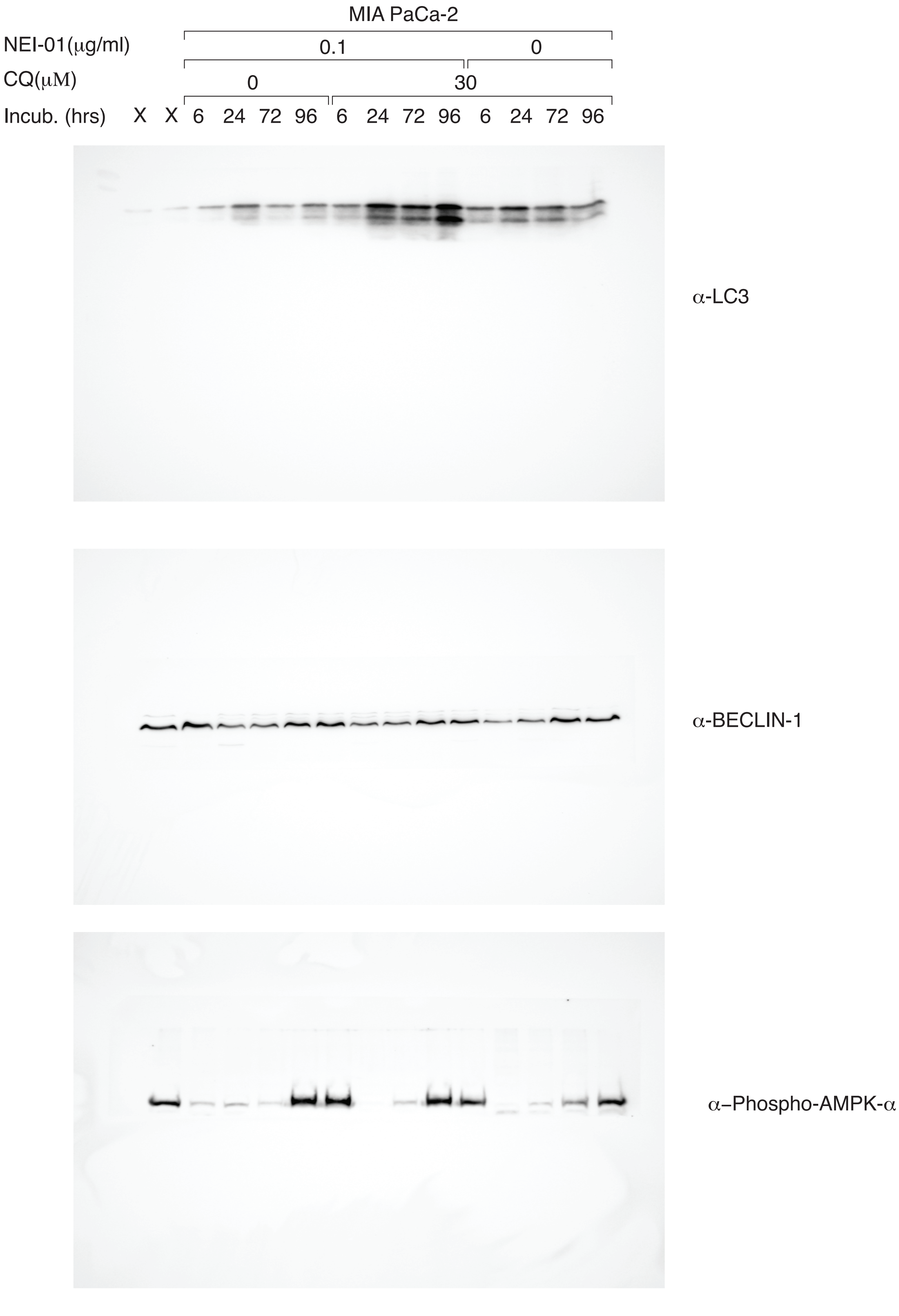
MIA PaCa-2
NEI-01(μg/ml)
0.1
0
CQ(μM)
0
30
Incub. (hrs)
X X 6 24 72 96 6 24 72 96 6 24 72 96
α-LC3
α-BECLIN-1
α–Phospho-AMPK-α

Fig 4C

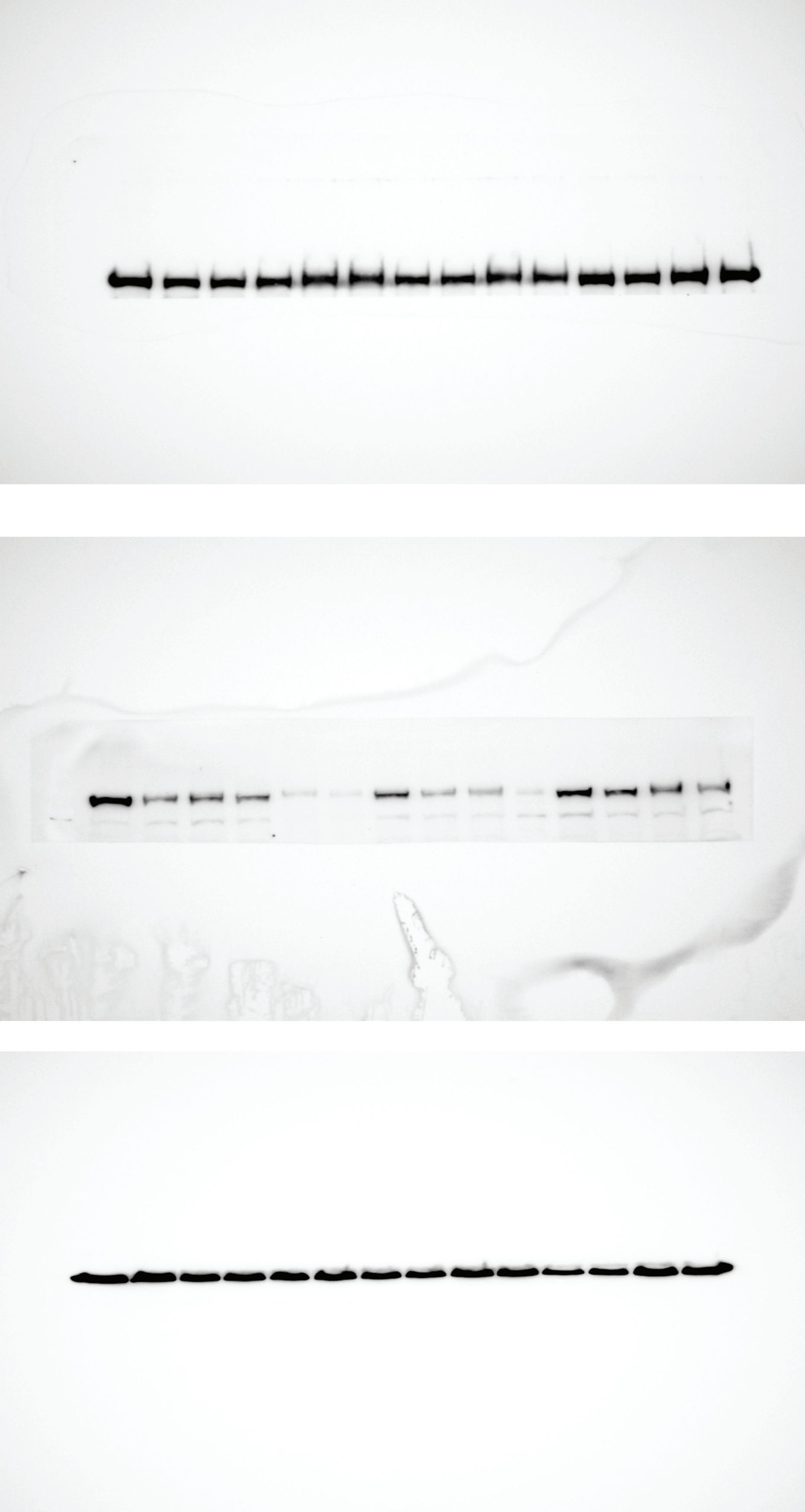

Fig 5C

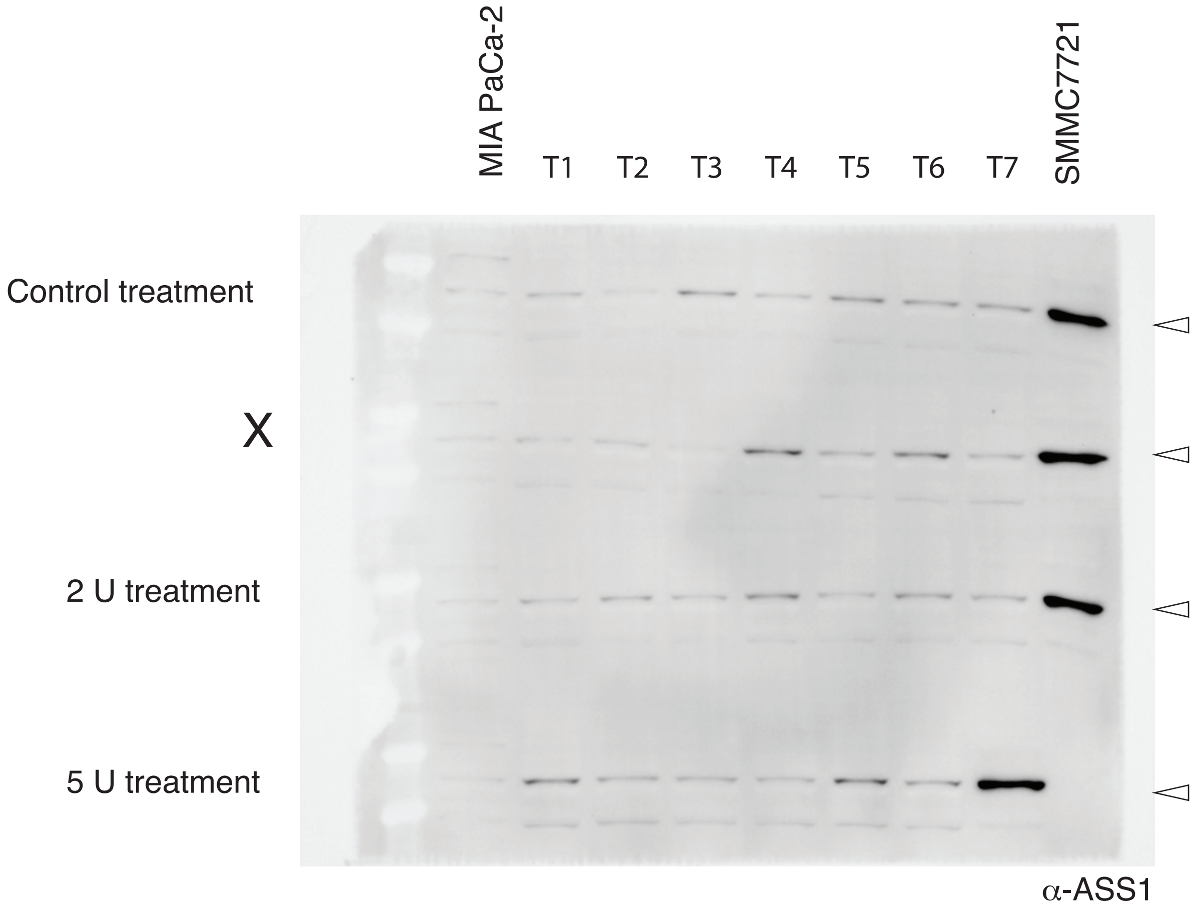

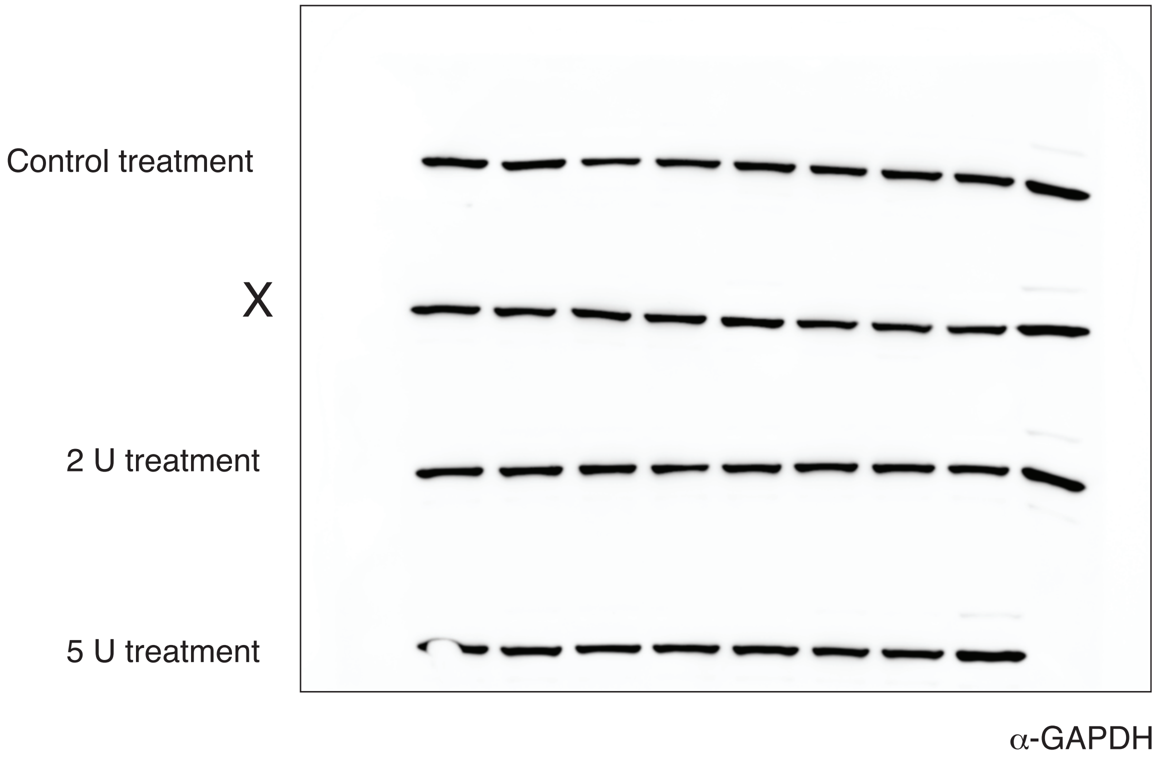

The images were not cropped or edited by any image processor.
After SDS-PAGE, the gels (4 in total) corresponding to the interested range of molecular size were cut and transfered to the same membrane for immunoblotting.

Fig 6A

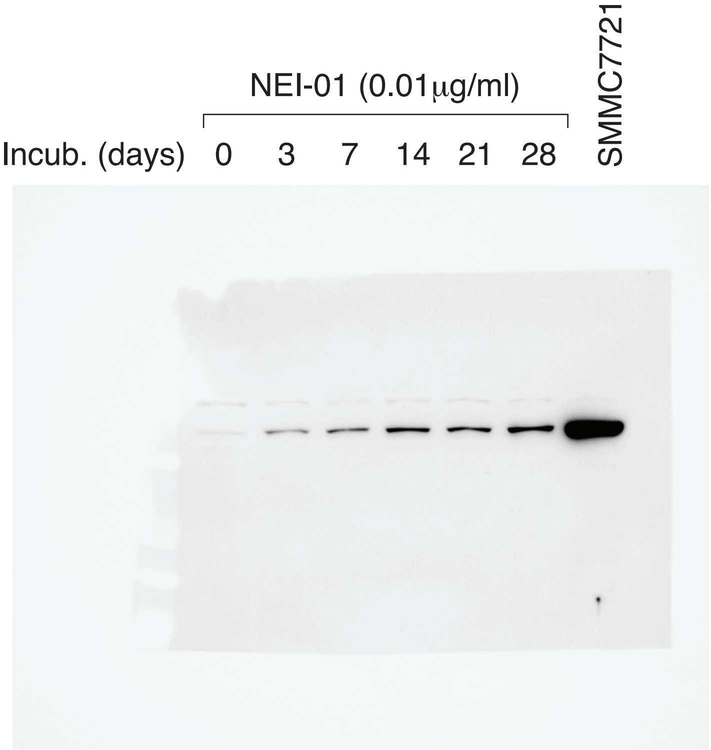

α-ASS1

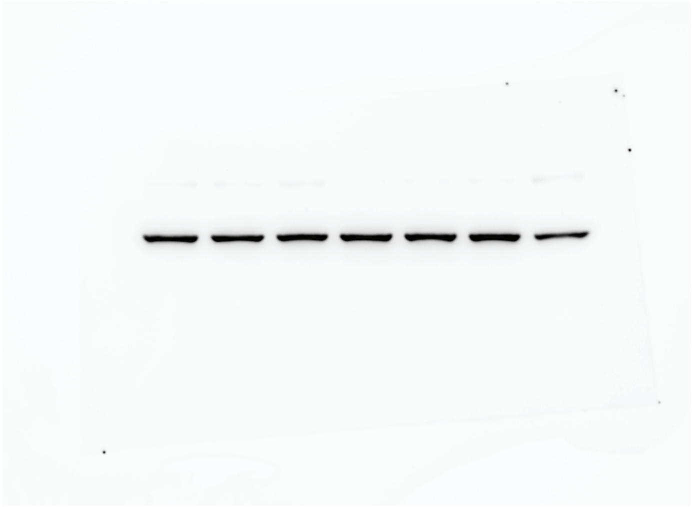

α-ACTIN

Fig 6B

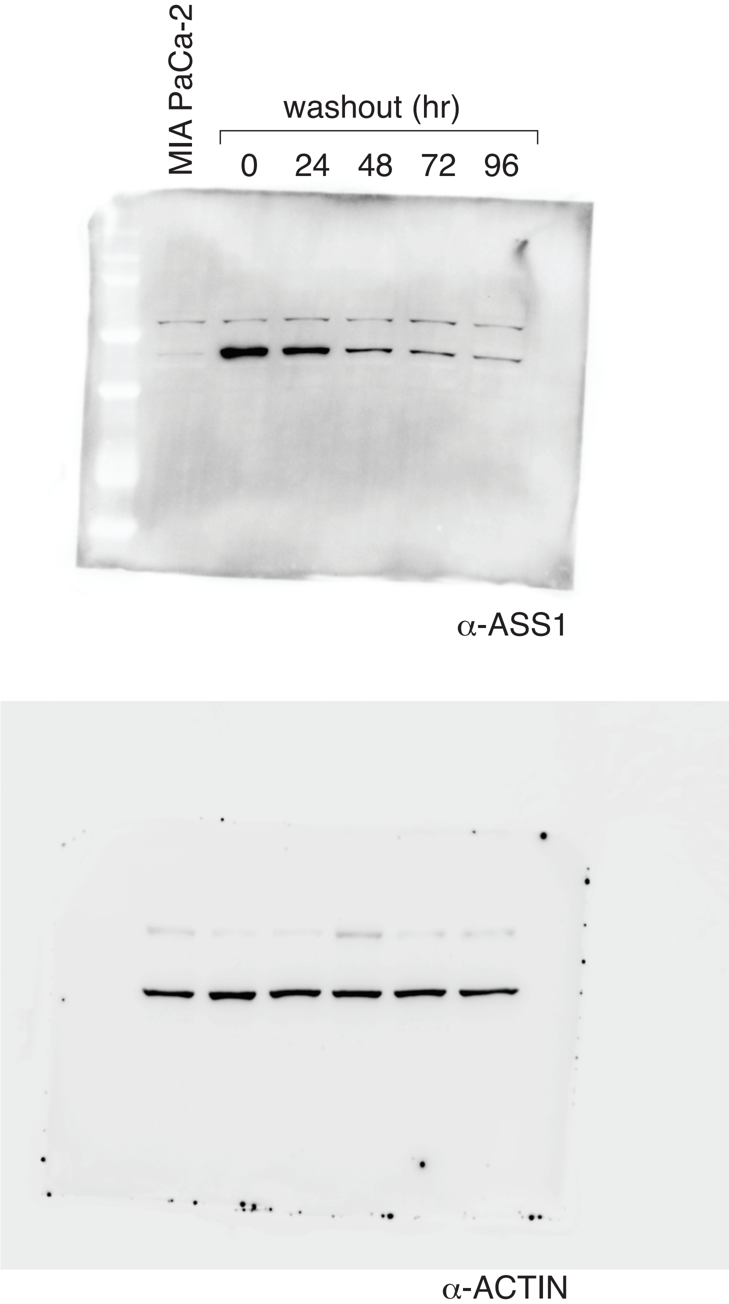

Supplement: S1 Raw images — (PDF) [file pone.0231633.s004.pdf]
